# Right choice, right time: Evaluation of an online decision aid for youth depression

**DOI:** 10.1111/hex.12510

**Published:** 2016-10-17

**Authors:** Magenta B. Simmons, Aurora Elmes, Joanne E. McKenzie, Lyndal Trevena, Sarah E. Hetrick

**Affiliations:** ^1^ Orygen The National Centre of Excellence in Youth Mental Health; ^2^ Centre for Youth Mental Health The University of Melbourne; ^3^ School of Public Health and Preventive Medicine Monash University Clayton Vic. Australia; ^4^ School of Public Health The University of Sydney Sydney NSW Australia

**Keywords:** adolescents, depression, patient decision aids, shared decision making, young adults

## Abstract

**Background:**

Appropriate treatment for youth depression is an important public health priority. Shared decision making has been recommended, yet no decision aids exist to facilitate this.

**Objectives:**

The main objective of this study was to evaluate an online decision aid for youth depression.

**Design:**

An uncontrolled cohort study with pre‐decision, immediately post‐decision and follow‐up measurements.

**Setting and Participants:**

Young people (n=66) aged 12‐25 years with mild, mild‐moderate or moderate‐severe depression were recruited from two enhanced primary care services.

**Intervention:**

Online decision aid with evidence communication, preference elicitation and decision support components.

**Main outcome measures:**

The main outcome measures were ability to make a decision; whether the decision was in line with clinical practice guidelines, personal preferences and values; decisional conflict; perceived involvement; satisfaction with decision; adherence; and depression scores at follow‐up.

**Results:**

After using the decision aid, clients were more likely to make a decision in line with guideline recommendations (93% vs 70%; *P*=.004), were more able to make a decision (97% vs 79%; *P*=.022), had significantly reduced decisional conflict (17.8 points lower (95% CI: 13.3‐22.9 points lower) on the Decisional Conflict Scale (range 0‐100)) and felt involved and satisfied with their decision. At follow‐up, clients had significantly reduced depression symptoms (2.7 points lower (95% CI: 1.3‐4.0 points lower) on the Patient Health Questionnaire nine‐item scale (range 0‐27)) and were adherent to 88% (95% CI: 82%‐94%) of treatment courses.

**Discussion and Conclusions:**

A decision aid for youth depression can help ensure evidence‐based, client‐centred care, promoting collaboration in this often difficult to engage population.

## 1 Background

Mental disorders are the leading cause of disease burden for young people in high‐income countries and the leading contributor to years of life lost to disability for young people aged 10‐24 worldwide, with unipolar major depression accounting for the greatest proportion of neuropsychiatric burden across multiple regions including Australia, the USA and the UK.^1^ In addition to being a major risk factor for suicide in adolescents, depression is also associated with poorer physical health, increased risk for substance abuse and comorbid mental disorders, such as anxiety, and poorer educational and vocational outcomes.^2^ Clinical practice guidelines (eg^3^, ^4^) detail the evidence‐based treatments available; however, a lack of implementation strategies for these guidelines, along with poor help‐seeking behaviours and clinical engagement, hampers the timely provision of appropriate treatment. To address these issues, as is recognized in these guidelines, evidence‐based client‐centred care that takes into account the effectiveness of treatment options as well as personal preferences and values is recommended.

There has been recent enthusiasm for the application of shared decision making (SDM) to the area of youth mental health,^5^, ^6^ including depression.^7^, ^8^ However, the rates of SDM in routine mental health care are likely to be low if adult data are any indication.^9^, ^10^,^11^, ^12^, ^13^ Decision aids can facilitate SDM and represent one way to approach this dilemma. Decision aids are evidence‐based tools that provide information about guideline concordant treatment options and invite users (clinician/s and client, plus caregiver/s where appropriate) to explore client values and preferences in relation to the treatment options. Decision aids have demonstrated effectiveness for decision‐related outcomes (eg reducing decisional conflict) in non‐psychiatric adult populations.^14^ In doing so, the information exchange between clinician and client is supported, and a treatment choice informed by evidence and individual client preferences can be chosen, which opens up the possibility of reducing decisional conflict, whilst at the same time increasing client satisfaction, engagement and adherence to the chosen treatment option.

There is an emerging body of work demonstrating the importance of SDM for treatment decisions for adults diagnosed with depressive disorders. In a recent randomized trial testing the effectiveness of a decision aid (in the format of “choice cards”) for antidepressant use, Le Blanc et al.^15^ found that the intervention resulted in greater knowledge, satisfaction and involvement in the decision and lower decisional conflict, without increasing the duration of the consultation time. This is supported by earlier work undertaken by Loh et al.,^16^ who conducted a randomized trial to test an online decision aid for adult depression. Participants using the decision aid were more satisfied and more involved in making the decision, and the use of the decision aid again did not increase consultation time. Additionally, the Quality Improvement in Depression study showed that higher levels of SDM resulted in higher levels of satisfaction^17^ and increased the likelihood of guideline concordant care and symptom reduction.^18^

In terms of youth depression, the Youth Partners‐In‐Care study enrolled young people aged 13‐21 years in a multisite‐randomized trial in the US comparing treatment as usual (n=207) with a quality improvement intervention (n=211) that included a SDM component (client and clinician treatment choice, with clinicians in the intervention group given information about client choice).^19^ At 6 months after enrolment in the study, young people in the intervention group had engaged in more treatment (including psychological therapies), were more satisfied with their care, had higher mental health‐related quality of life and had significantly reduced symptoms of depression. There were long‐term benefits, including an indirect intervention effect on depression severity, found at 12‐ and 18‐month follow‐up time points.^20^ Due to the complex nature of the intervention, it is not possible to differentiate any effects of the client choice component. Although this study demonstrates the potential for SDM to improve outcomes for youths with depression, there is a lack of SDM tools (eg decision aids) in this area.^14^, ^21^ The only decision aids we located in youth mental health were for parents as decision makers for their children (eg ADHD^22^).

This gap in decision support needs careful attention. Individuals faced with decisions about treatment for mental ill‐health are likely to require a range of information to fully engage in SDM;^23^ young people facing these decisions need specialized support given the specific evidence to consider (eg harms and benefits of antidepressant medication^24^, ^25^) and the developmental stages they move through during this time, including taking increasing responsibility for their own mental health. In earlier work, we demonstrated the complex nature of decision making in youth depression. Qualitative interviews with clinicians^26^ and clients and caregivers^8^ found that processes (eg information provision) as well as interpersonal factors (eg trust) were important and that whilst a SDM model was almost unanimously endorsed by each group, there was a lack of available tools to facilitate SDM. To address this, a prototype decision aid was developed to support treatment decision making for young people with moderate‐severe depression and was piloted with five young people.^27^ Although these results were favourable, the decision aid provided support for only one level of depression severity.

## 2 Aim

The aim of this study was to develop and evaluate an online decision aid for young people experiencing mild, mild‐moderate or moderate‐severe depression.

### 2.1 Research questions

- Does a decision aid help young people to make a decision, one that is in line with both the evidence and their personal preferences and values?
- Does a decision aid help young people to feel involved in the decision‐making process, feel satisfied with their decision and feel less confused about what to do?
- After using a decision aid do young people stick with their original decision, are they adherent to the treatment plan and do they have reduced symptoms of depression?
- After using a decision aid are clinicians satisfied with the decision?

### 2.2 Design

An uncontrolled cohort study with pre‐decision, immediately post‐decision and follow‐up measurements.

### 2.3 Intervention

The “youth depression decision aid” was on online website designed to be used by both clients and clinicians, including together in clinical appointments, and with caregivers where appropriate. A full description of the tool is shown in Box 1 . In summary, the main sections of the decision aid were as follows: (i) “What matters to you?”, (ii) “Treatment options”, (iii) “Your decision”, and (iv) “Information”. No modifications were made to the decision aid during the evaluation phase.

Box 1 **Description of the content of the online decision aid**

1

**Table:** 

Section of the online decision aid	Description
1. Mood questionnaire (PHQ‐9; completed in the waiting room before the decision appointment)	All participants completed the PHQ‐9, the results of which were shared with the clinician.
2. “What Matters to You?” (completed in the waiting room before the decision appointment)	All participants completed this section, which was designed to elicit personal needs, preferences and values around different treatment options for depression. The results of this were shared with the clinician in the decision appointment and were revisited after section Design (“Treatment Options”) was viewed and discussed.
3. “Treatment Options” (Viewed in the decision appointment to facilitate conversation about evidence and client preferences between the client and clinician)	Depending on mood severity (based on results of PHQ‐9 and clinician assessment), participants viewed one of three sections of the decision aid (3a, 3b or 3c).
a. Mild depression: “Should I make lifestyle changes or use guided self‐help?”	Provided details of lifestyle changes (eg fact sheets on healthy eating, exercise, sleep hygiene) and comparison of guided self‐help options (e‐mental health programs such as MoodGYM^a^, E‐Couch^a^ and ReachOut Central^b^).
b. Mild‐moderate depression: “Should I undertake cognitive behavioural therapy or not?”	Provided details of CBT (including fact sheet) and comparison of CBT vs no treatment, including a graph^c^^,^ d depicting chance of “getting better” (remission) within 12 weeks.
c. Moderate‐severe depression: “Should I take antidepressant medication in addition to cognitive behavioural therapy?”	Provided details of CBT and SSRI medication (including fact sheets) and comparison between CBT vs CBT plus fluoxetine, including a graph^c^^,^ d depicting chance of “getting better” (remission) within 12 weeks and a graph depicting chance of experiencing suicidal thoughts and behaviours within 12 weeks.
4. “Your Decision” (completed with the clinician in the decision appointment)	All participants completed this after viewing Section Design. Based on the Ottawa Personal Decision Guide^e^, known causes of decisional conflict were listed for discussion. Participants were asked whether they had enough knowledge; were clear about their values; felt they had enough support and advice and were clear about their choice. A date was entered for when the decision would be revisited. If the participant was not ready to decide, then a date for their next appointment was entered.
5. “Information”	All participants had access to a section of the decision aid that provided information about depression and treatment, including fact sheets, websites, videos and audio recordings. These could be viewed before, during or after their appointment.

a Bennett K, Reynolds J, Christensen H, Griffiths KM. e‐hub: an online self‐help mental health service in the community. *Med J Aust*. 2010;192(11 Suppl):S48‐52.

b Burns JM, Webb M, Durkin LA, Hickie IB. Reach Out Central: a serious game designed to engage young men to improve mental health and wellbeing. *Med J Aust*. 2010;192(11 Suppl):S27‐30.

c NB: All graphs were developed in line with the International Patient Decision Aids Standards.

d Trevena LJ, Zikmund‐Fisher BJ, Edwards A, Gaissmaier W, Galesic M, Han PKL, et al. Presenting quantitative information about decision outcomes: a risk communication primer for patient decision aid developers. *BMC Med Inform Decis Mak*. 2013;13(Suppl 2):S7.

e O'Connor A, Stacey D, Jacobsen MJ. Ottawa Personal Decision Guide Ottawa, Canada: Ottawa Hospital Research Institute and University of Ottawa; 2016 [cited 2016 11th July 2016]. Available from: https://decisionaid.ohri.ca/decguide.html.

The decision aid was developed in line with the International Patient Decision Aids Standards (IPDAS;^28^, ^29^) and the Ottawa Decision Support Framework (ODSF;^30^), ensuring that the development process was rigorous and underpinned by key theories related to decision making.^31^ A working group was established that included experts in youth depression (SH), shared decision making (LT), youth shared decision making (MS) and biostatistics (JM).

A number of decisions were made by the working group to determine the way in which evidence was translated for the decision aid. For example, although there is evidence that both cognitive behavioural therapy (CBT) and interpersonal therapy are effective for the treatment of youth depression which is reflected in the clinical practice guideline recommendations,^3^ we chose to present information on CBT alone due to the higher availability of CBT (in both person and online via a national counselling service eheadspace^32^) both in the services where the study was set and in Australia more broadly as we wanted to ensure relevance for future dissemination. In terms of the levels of evidence available for each depression severity, different approaches were taken for the evidence communication sections of each decision. For moderate‐to‐severe depression, there was sufficient evidence in existing systematic reviews^25^, ^33^ to develop the decision aid. For mild‐moderate depression, although there was adequate high‐quality evidence (ie from randomized trials), there was no current systematic review. To fill this gap, our broader research team undertook a systematic review of the effectiveness of psychological therapies in youth depression (manuscript in preparation). For mild depression, it was deemed that there was insufficient high‐quality evidence (ie from randomized trials) to use for this decision and so instead we presented key information (eg purpose, length and duration of intervention; target audience; nature of content) for each different option in a comparable way.

We consulted with consumers, caregivers and clinicians throughout the development process, who provided feedback about the design, content (including language used in the tool), format and function of the tool as well as how the tool was used in clinical practice. These groups included The Platform team,^34^ Family Peer Support Officers and Youth Mood Clinic staff members from Orygen Youth Health and Youth Advisory Groups, private practitioners and intake workers from headspace Glenroy and Craigieburn. Previous research underpinning the current decision aid also involved these three groups, including both consultation and formal research interviews.^8^, ^26^, ^27^ The decision aid is not yet publicly available as we intent to undertake a larger study to test the effectiveness in the context of a randomized trial in the future.

### 2.4 Evaluation of the decision aid

#### 2.4.1 Setting

The evaluation of the decision aid was undertaken at two headspace centres which are enhanced primary care services with a focus on youth mental health for young people aged 12‐25 years in the northern suburbs of Melbourne, Australia (Glenroy and Craigieburn).^35^ headspace centres have a focus on early intervention; approximately 75% of mental disorders emerge before the age of 25 years,^36^ and the age range of the service reflects the importance of effective treatment during this time.

#### 2.4.2 Sample

The inclusion criteria for clients were (i) current “headspace” client, (ii) scored 5 or more on the Patient Health Questionnaire (PHQ‐9),^37^ (iii) were able to use the decision aid (no visual impairment, sufficient English language skills), and (iv) provided informed consent. The inclusion criteria for clinicians were (i) willing to use the decision aid with their client, and (ii) provided informed consent.

#### 2.4.3 Procedure

**Figure 1:**
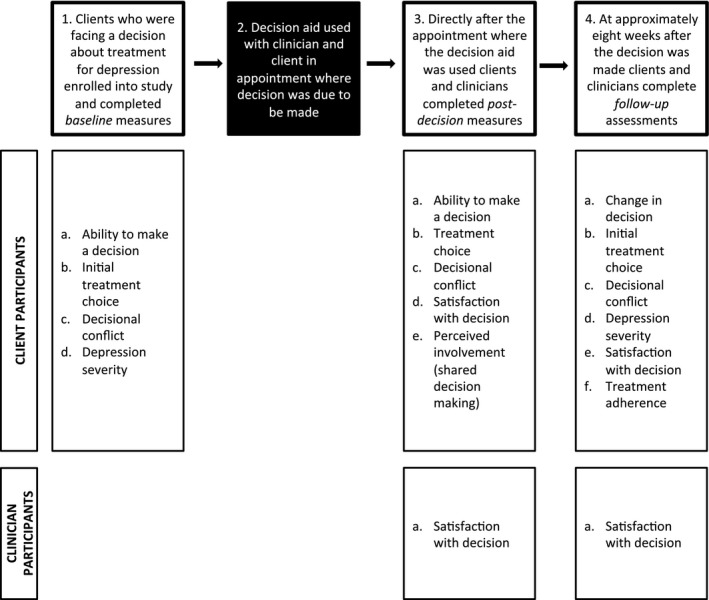
Assessment time points and related assessments for client and clinician participants

Recruitment took place between July 2014 and May 2015 inclusive. Clinician participants were recruited during and after staff meetings. Clinician participants were provided with an instructional session to build confidence in using the decision aid; however, the decision aid was designed for use as a “stand‐alone” tool. No further training was provided to clinicians. The decision aid website was made available to clients and clinicians on tablet computers. Client participants were identified by clinicians and referred to the study. Two randomized trials^38^, ^39^ were also recruiting participants with moderate‐to‐severe depression during this period, and so referrals included any headspace client with depressive symptoms who did not meet inclusion criteria for these larger studies (eg mild depression) or who declined participation but consented to be approached about other research studies. It is also possible that clinicians declined to pass on information of a client they felt would not be suitable for research (eg in current state of crisis). After referral, those who provided informed consent to participate undertook a baseline assessment (Figure 1). Following this, the decision aid was used during their next appointment (which sometimes occurred on the same day as, but after, the baseline assessment) at the conclusion of which the decision assessment was performed. No formal training was provided to client participants as the tool was designed for use without assistance. The researcher was present whilst the client completed the questionnaires in either the waiting room or a consultation room for privacy; however, no assistance was necessary. The decision aid was designed for use in the existing standard 50‐minute appointment where a decision was being made. There were no instances where additional appointments had to be scheduled. Although clients were able to use the decision aid with caregivers if they were present, this did not eventuate. The researcher was not present in the decision assessment, and there was no formal assessment of fidelity; however, all participants were asked which pages of the website they visited to ensure that at least the first three sections were used. Participants were contacted at approximately 6 weeks after their decision assessment to complete the follow‐up assessment measures. Clinician participants also completed measures at the decision and follow‐up time points.

#### 2.4.4 Follow‐up assessments

The original aim was to follow up client and clinician participants between 6 and 8 weeks after the decision aid was used. This time point was chosen to maximize participant retention. The mean number of appointments attended by headspace clients for mental health problems nationally is 4.4.^40^ In instances where the participants were unable to complete follow‐up assessments within this time frame, assessments were still conducted and the time between decision and follow‐up assessments was recorded. Alternatively, if a person was unable to complete the follow‐up assessment after the required time, they were seen as soon as they were available and asked to reflect on the 6‐8 weeks after the decision was made. The time to follow up was most affected during holiday periods, resulting in a range of 5‐28 weeks (mean (M)=8.3; standard deviation (SD)=4.19).

#### 2.4.5 Measures

Client participants were asked to state whether or not they felt able to make a decision about what treatment to choose before and then again after using the decision aid, when they were also asked whether the decision was in line with their preferences and values. At follow‐up (approximately 8 weeks later), they were asked whether this decision had changed and if so why. These responses, along with items relating to previous treatment history, were used to assess whether or not the decisions being made were in line with the clinical practice guidelines for the treatment of youth depression.^3^ Examples of decisions that were deemed non‐guideline concordant include the following: (i) client participants with mild depression who opted for antidepressant medication (with or without CBT), (ii) client participants with any depression severity who opted for medication alone (without trying CBT first), and (iii) client participants with moderate‐severe depression who opted for lifestyle changes and/or guided self‐help.

Decisional conflict was measured with the Decisional Conflict Scale (DCS)^41^ which was previously piloted with a small number of young people.^27^ The DCS is a 16‐item measure that uses a 0‐4 Likert scale. It has a total score range of 0‐100, where higher scores indicate higher decisional conflict (undesired outcome). Perceived involvement (shared decision making) in the decision‐making process was measured using the Shared Decision Making Questionnaire (SDMQ).^42^ The SDMQ is an 11‐item self‐report questionnaire with 4‐point Likert responses,^1^, ^2^, ^3^, ^4^ resulting in a minimum score of 11 and a maximum score of 44. How satisfied participants were with the decision was measured with the Satisfaction With Decision (SWD) scale,^43^ a six‐item 1‐5 Likert scale self‐report questionnaire with a maximum score of 30 where higher scores indicate higher satisfaction with the decision.

At follow‐up, client participants were asked three investigator‐devised open‐text questions specific to each treatment type they had chosen (guided self‐help and lifestyle options, CBT and antidepressant medication). For example, participants who had undertaken CBT were asked the following: “How regularly have you been attending appointments? (eg weekly, fortnightly, monthly)”; “For how long have you been doing this therapy?” and “Have you missed any scheduled appointments? If so, how many?”. Responses were then coded in terms of frequency and adherence. Adherence was categorized into full adherence; minor non‐adherence (80% or more sessions attended); moderate non‐adherence (50%‐79% of sessions attended) and non‐adherence (<50% of sessions attended). Depression severity was measured using the nine‐item version of the self‐report Patient Health Questionnaire (PHQ‐9) 37 which has previously been validated in an adolescent sample.^44^ Each item is measured using a Likert scale from 0 to 3 resulting in a maximum score of 27, which indicates higher depression severity. Respondents rate their symptoms based on the 2‐week period prior to completing the questionnaire. All measures were reviewed by a youth advisory group prior to the study commencing.

### 2.5 Statistical analysis

We present frequencies and percentages of responses to binary variables, and means, standard deviations, and nonparametric statistics for continuous outcomes. McNemar's test was used to compare changes in paired binary variables over time. Paired *t*‐tests were used to test for change in continuous outcomes over time, and independent sample *t*‐tests were used to compare mean scores between groups. For the clinician‐rated satisfaction with the decision at follow‐up and at 6 weeks, the confidence interval for average satisfaction was calculated using robust cluster standard errors that allow for correlation of responses arising from the clinician treating multiple clients. Statistical analyses were undertaken in SPSS version 22.0^45^ and Stata version 14.^46^ We aimed to recruit 64 clients, a number which was selected for feasibility reasons (ie time and available funding).

### 2.6 Ethics

This study received ethics approval from the University of Melbourne Behavioural Sciences Human Ethics Sub‐Committee (reference number 1339306) and conforms to the provisions of the Declaration of Helsinki (as revised in Tokyo 2004), available at http://www.wma.net/en/20activities/10ethics/index.html.

## 3 Results

### 3.1 Participant descriptives

**Figure 2:**
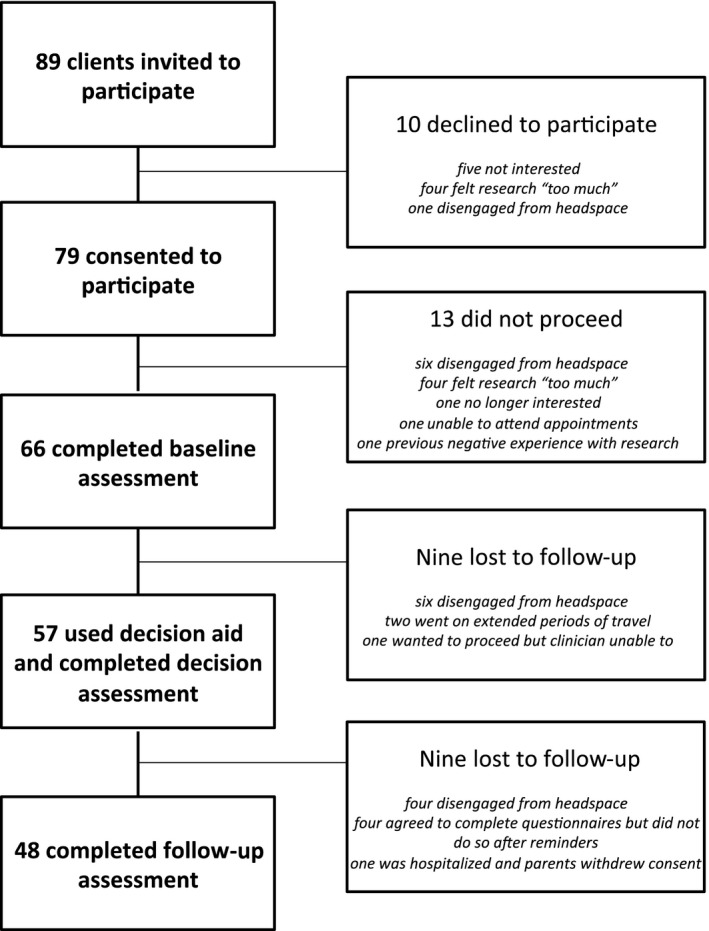
Recruitment rates and reasons for attrition

Of the 89 clients invited to participate, 66 clients (74%) provided informed consent to participate in the study. Of these 66, 57 (86%) used the decision aid and completed post‐decision assessments and 48 (73%) completed the follow‐up assessment (Figure 2). There were no statistically significant or important differences in the available demographic and clinical characteristics between participants who did and did not complete the follow‐up assessment (Table 1).

**Table 1:** Demographic and depression severity details of participants who did and did not complete the follow‐up assessment

	All participants (n=66)	Participants who completed follow‐up assessment (n=48)	Participants who did not complete a follow‐up assessment (n=18)	*P* a
Gender N (%)				
Female	54 (81.8)	38 (79.2)	16 (88.9)	.611
Male	11 (16.7)	9 (18.7)	2 (11.1)
Other	1 (1.5)	1 (2.1)	000 (00.0)
Age in years M (SD)	18.5 (3.42)	18.42 (3.45)	18.8 (3.40)	.914
Baseline PHQ‐9 Score M (SD)	13.95 (5.58)	14.0 (5.22)	13.7 (6.60)	.129

a Independent *t*‐tests were used to compare age and PHQ‐9 scores, and the chi‐square test was used to compare gender between participants who completed and did not complete follow‐up.

Client participants were aged between 13 and 25 years with a mean age of 18.5 years (SD=3.42). Fifty‐four (82%) identified as female; 11 (17%) identified as male (including one participant who identified as a transgender male), and one participant (2%) identified as demigender (partially identified as female). In terms of depression severity, 12 (18%) had PHQ‐9 scores suggesting mild depression, 17 (26%) mild‐moderate depression and 37 (56%) moderate‐severe depression. Thirty‐five participants (53%) had previously received treatment for depression: 10 (16%) had received counselling (eg CBT); 3 (5%) had taken medication; 15 (24%) had tried both counselling and medication and 4 (6%) had tried something else (herbal medication, music, “squeezing ice (and) writing on myself” and “inpatient service”).

In total, 23 clinicians were invited to participate and took part in the study. Of these, 20 (87%) were female and 3 (13%) were male. In terms of professional background and role, six were working in the Youth Access Team (five registered or clinical psychologists and one provisional psychologist); 16 were working as private practitioners (14 registered or clinical psychologists, two occupational therapists) and one general practitioner. Clinician participants used the decision aid with between one and nine clients, with the median number of times being 2 (IQR=1‐3.5).

### 3.2 Were clients able to make a decision and was it in line with both the evidence and personal preferences and values?

Client participants were asked whether they had made a decision about treatment before and then again after using the decision aid. Clients were more likely to make a decision that was guideline concordant after using the decision aid (93%) than before (70%) (McNemar test *P*=.004). Clients were significantly more able to make a decision after using the decision aid (97%) than before (79%) (McNemar test *P*=.022). Client participants who were able to make a decision were asked if the chosen treatment was the one that they most preferred and if it matched their personal needs and preferences; 53 (100%) of clients endorsed both of these items.

### 3.3 Did clients feel involved in making the decision, were they satisfied with the decision and did they feel less confused about what to do?

Scores on the SDMQ ranged from 29 to 44 with a mean score of 37.4 (SD=4.30), indicating high levels of perceived involvement in making a decision about their own treatment. Scores on the SWD scale ranged from 16 to 30, with a mean score of 25.8 (SD=3.14), indicating high levels of satisfaction especially given those who were unable to make a decision also completed this scale. There was a mean reduction in DCS scores between baseline (M=37.9) and after using the decision aid (M=21.1) of 17.8 (95% CI [13.25, 22.94], *P*<.001), representing a statistically significant decrease in decisional conflict. This means that participants felt less conflicted about which treatment option to choose after using the decision aid.

### 3.4 Were clinicians satisfied with the decision?

Clinician participants (n=21) were also asked to rate how satisfied they were with the decision and did so for 49 decisions. The interquartile range was 24‐28, with a mean score of 25.4 (95% CI: 23.7‐27.1), also indicating high levels of satisfaction.

### 3.5 Did the treatment change during the follow‐up period, were clients adherent to their chosen treatment option and did their depression scores improve?

At follow‐up, there were clinician‐rated data for 46 client participants. Of these, 35 (76%) were still engaged in the original treatment choice. Of the 11 clients who had changed their treatment, five had not engaged in the treatment that was initially decided upon; one had stopped because they were “better”; one was in crisis too often to undertake psychological therapy; one had gone on to develop first‐episode psychosis, and treatment had changed in line with this; one had over‐reported depressive symptoms at the time of making a decision, and so treatment had been revised in line with this (to a guideline concordant treatment option); and one client had required family therapy, but the family had failed to engage.

Client‐reported adherence data were available for 47 participants. Due to the fact that some clients engaged in more than one treatment, adherence data were collected for 89 “courses” of treatment (27 courses of lifestyle changes or guided self‐help; 45 courses of CBT and 17 courses of medication); however, data were missing in seven courses, and so we present data for 82 “courses” of treatment. For lifestyle changes and guided self‐help, 18/22 (82%) of client participants reported engaging in these activities on a daily, weekly or fortnightly basis, and 4/22 (18%) reported moderate non‐adherence. For CBT, 35/43 (81%) of client participants had weekly to fortnightly sessions; 3/43 (7%) had fortnightly to monthly sessions and 5/43 (12%) had moderate non‐adherence. For medication, 16/17 (94%) of participants had full adherence or minor non‐adherence to daily doses. Only one client had ceased medication during the follow‐up period. The subgroup of clients who reported adherence data for medication included a mix those who chose medication (in addition to CBT; n=13) at the time of using the decision aid and four participants who had not (three of whom reported taking medication before using the decision aid and one of whom commenced medication during the follow‐up period). Follow‐up data were not available for two clients who had chosen medication and CBT after using the decision aid.

At follow‐up, clients had significantly reduced depression symptoms compared with baseline (2.7 points lower (95% CI: 1.3‐4.0 points lower) on the PHQ‐9).

### 3.6 Were clients and clinicians still satisfied with the decision that had been made?

At follow‐up, client and clinician participants were asked to rate how satisfied they were with the decision that had been made at the time of using the decision aid. There were SWD data for 45 clients, with an interquartile range of 22‐34 and mean score of 26 (95% CI: 24.7‐27.1). Clinician participants (n=21) rated a total of 45 decisions, with an interquartile range of 23‐29, and mean score of 25.3 (95% CI: 23.9‐26.8). This indicates that both clients and clinicians were still very satisfied with the decision‐making processes at approximately 8 weeks after the decision was made.

## 4 Discussion

The results of this study confirmed that it is possible to support client‐centred care and collaborative decision making when treating youth depression. After using the decision aid, young people were more able to make a decision and were more likely to choose a treatment in line with the evidence. They felt involved in making the decision, reported that it was in line with their values and preferences, were satisfied with the decision and had significantly reduced decisional conflict. At approximately 8 weeks after making the decision, they had significantly reduced depression scores and more than 80% of participants were adherent with their treatment choice. This is of particular importance given that non‐adherence rates are usually high in this population.^47^

A major strength of this study was the development process of the decision aid, which involved the most relevant and up‐to‐date evidence and the inclusion of relevant stakeholders (eg professional experts and consumer experts). The main limitation of this study is that there was no control group. We can therefore not attribute the observed changes in outcomes such as depression to the decision aid. Due to the very novel nature of the study in terms of shared decision making in youth mental health, we felt that a “proof of concept” study was an appropriate starting point to determine the feasibility and acceptability of the intervention. It is possible that the change in decisions (from non‐guideline concordant to guideline concordant) was due to the information the clinicians would have provided anyway. A randomized trial that not only compares the decision aid with decision making as usual but also includes fidelity checks of the intervention and control groups would help determine this. Another limitation is that we do not have adherence data for the nine participants who did not complete a follow‐up assessment, and it is possible that these lost data represent higher rates of non‐adherence, particularly the four participants who had disengaged from the service altogether. Two of the measures (SDMQ and SWD) had not previously been validated in youth settings; however, they were both reviewed by youth reference group members prior to use. Lastly, there is a possibility that some selection bias arose because (i) clinicians passed on referrals for potential client participants, and (ii) recruitment preference was given to two larger clinical trials meaning that not all clients experiencing symptoms of depression could be approached.

The results of this study contribute to a number of fields of research, including the emerging field of youth shared decision making.^5^, ^6^ For those concerned with the ethical issues of appropriate treatment of youth depression, the decision aid offers a way to communicate the evidence in terms of both effectiveness (ie potential benefits) and the evidence for potential harms. For example, although the absolute risk of suicidal ideation and behaviours as a side‐effect of antidepressant medication for youth is small, it represents an approximate doubling of relative risk.^24^, ^25^ The risk communication section of the decision aid included the provision of this information, allowing for a collaborative discussion between client and clinician about the importance of monitoring both mood symptoms and suicidal ideation and behaviours, an issue of concern to health practitioners.^48^

For services, the decision aid offers a prospective implementation strategy for translating high‐quality evidence into everyday practice whilst ensuring client‐centred care at the same time. Involving people in making decisions about their own mental health care is advocated for in clinical practice guidelines (eg^3^, ^4^) and has been a centrepiece of policy‐related reform both locally (eg^49^) and internationally.^50^ Without interventions to facilitate shared decision making, there is no formal way to ensure that this occurs in day‐to‐day practice. The full implementation of evidence‐based practices, including the use of clinical practice guidelines, remains a major challenge,^51^ yet the data from this study suggest that the use of a decision aid may improve concordance with clinical practice guidelines.

For youth mental health especially, it is imperative that these interventions involve online technologies as both young people and their clinicians emphasize the importance of technology in managing mental health and well‐being.^52^ A recent review highlighted the lack of online tools designed to be used during the consultation, and this decision aid provides an example of one way in which this gap can be addressed.^53^

### 4.1 Further research

To fully understand the effectiveness of the decision aid, there is a need to test its effectiveness in a randomized trial designed in line with appropriate implementation frameworks.^54^ In designing such a trial, careful consideration should be given to ensuring fidelity of the intervention (ie objectively measuring SDM) and collecting outcomes along the causal pathway that may help to explain the observed effects. Understanding how a decision aid fits with on‐going care (eg the monitoring of depressive symptoms and suicidal ideation, additional clinical engagement and adherence strategies) and an exploration of implementation‐related barriers and enablers would maximize the chance of uptake in a variety of service settings. Including different types of psychological therapy where available may make the decision aid more relevant for services that provide modes other than CBT. More detailed consideration of age‐related and developmental factors, the involvement of caregivers and comorbid mental disorders are also candidates for related future exploratory research.

## 5 Conclusions

Supporting young people to make decisions about treatment for depression that are based on both evidence and personal preferences and values offers the best chance for matching them with the right treatment at the right time. Connecting young people with effective treatment in a timely manner can help to ease the burden of depressive symptoms experienced during a depressive episode and minimize the potential negative aspects of depression, such as a decline in social and occupational functioning, or relapse. Providing a positive decision‐making experience also offers a chance to engage this vulnerable, and traditionally tenuously engaged, group. Facilitating shared decision making at this age not only provides the best chance for the young person to improve their mental health, but also teaches them key skills in how to make informed decisions about their own care that can be used in future decisions about mental and general physical health. The results from this study demonstrate a proof that the concept of shared decision making for young people with mental ill‐health, whereby they are the primary decision makers, is feasible and may increase key factors related to clinical engagement and adherence to treatment.

## Conflict of Interest

The authors have not conflict of interests to declare.
